# Significantly improved solvent tolerance of *Escherichia coli* by global transcription machinery engineering

**DOI:** 10.1186/s12934-015-0368-4

**Published:** 2015-11-05

**Authors:** Fa Zhang, Xiaohong Qian, Haiming Si, Guochao Xu, Ruizhi Han, Ye Ni

**Affiliations:** The Key Laboratory of Industrial Biotechnology, Ministry of Education, School of Biotechnology, Jiangnan University, 214122 Wuxi, Jiangsu China

**Keywords:** Global transcription machinery engineering, Sigma factor 70, Organic solvent tolerance, *Escherichia coli*, *gapA*, *sdhB*, *pepB*

## Abstract

**Background:**

*Escherichia coli* has emerged as a promising platform microorganism to produce biofuels and fine chemicals of industrial interests. Certain obstacles however remain to be overcome, among which organic-solvent tolerance is a crucial one.

**Results:**

We used global transcription machinery engineering (gTME) to improve the organic-solvent tolerance (OST) of *E. coli* JM109. A mutant library of σ^70^ encoded by *rpoD* was screened under cyclohexane pressure. *E. coli* JM109 strain harboring σ^70^ mutant C9 was identified with capability of tolerating 69 % cyclohexane. The *rpoD* mutant contains three amino-acid substitutes and a stop-codon mutation, resulting a truncated sequence containing regions σ^1.1^ and σ^1.2^. Total protein difference produced by *E. coli* JM109 strain harboring C9 was examined with 2D-PAGE, and 204 high-abundant proteins showed over twofold variation under different solvent stress.

**Conclusions:**

Our results show that several genes (*gapA*, *sdhB*, *pepB* and *dppA*) play critical roles in enhanced solvent tolerance of *E. coli*, mainly involving in maintaining higher intracellular energy level under solvent stress. Global transcription machinery engineering is therefore a feasible and efficient approach for engineering strain with enhanced OST-phenotype.

**Electronic supplementary material:**

The online version of this article (doi:10.1186/s12934-015-0368-4) contains supplementary material, which is available to authorized users.

## Background

The increasing attention to green chemistry has prompted the production of non-renewable fuels, materials, pharmaceuticals, and fine chemicals by microbial factories [1]. *Escherichia coli* as one of the most important platform microorganisms, could be applied as a whole-cell biocatalyst, which provides safe intracellular environment for enzymes [2]. In whole-cell biocatalysis, nonaqueous system (such as organic solvents) is often adopted to facilitate the solubility of hydrophobic substrates and (or) products [3]. Organic solvents are toxic to most microorganisms. *E. coli* was reported to barely tolerate organic solvents with Log*P* values lower than 3.4–3.8 [4]. For example, toluene is toxic to *E. coli* cells even at concentrations as low as 0.1 % [5]. Hence, it is of great importance to develop organic-solvent tolerant (OST) *E. coli* strains for industrial applications.

Since the first toluene tolerant strain *Pseudomonas putida* IH-2000 identified in 1989, extensively work had been focused on *P. putida* and *Clostridium* species etc. [6, 7]. Various OST mechanisms have been proposed, including cell membrane adaptations [8], cell morphology [9], and efflux pumps etc. [10, 11]. Traditional strain engineering methods, such as adaptation [12], enrichment cultivation [13], chemical and physical mutagenesis [14], have been widely used for developing OST strains. Global transcription machinery engineering (gTME) is a novel directed evolution strategy to assist in unlocking complex phenotypes by disturbing the transcription at genome level. Alper and co-workers obtained yeast strains that tolerated ethanol up to 20 % (v/v) [15]. In the past few years, a number of gTME-aided studies have outperformed those of traditional methods, resulting desired phenotypes more effectively. Several transcription factors, such as sigma factor in bacteria [16], zinc finger-containing artificial transcription factor [17], *Spt15* in yeast [18] were widely used as a potential tool to improve strain tolerance and increase biofilm formation. Sigma factor 70 (σ^70^) is the most common transcription factor in *E. coli*. In addition to binding to RNA polymerase and recognizing DNA template strand, it can also alter the affinity of RNA polymerase to the promoter. Most importantly, the transcriptional efficiency can be regulated by mutation of σ factor [19, 20]. Alper and Stephanopoulos successfully constructed an *E. coli* strain that could tolerate as high as 60 g/L ethanol by mutating *rpoD* [21]. By random mutagenesis of *rpoD* and *rpoS*, Yu and coworkers obtained an *E. coli* mutant that could produce 561.4 mg/L hyaluronic acid [22].

In our previous study, an OST *P. putida* JUCT1 that tolerated 60 % cyclohexane was obtained by gradient adaptation. Based on two-dimensional electrophoresis (2-DE), two 3-hydroxyacid dehydrogenase family genes, *mmsB* (from *P. putida*) and *zwf* (from *E. coli*), were identified and proved to be critical for the enhanced solvent tolerance in both *P. putida* and *E. coli* [23, 24].

In this study, we evaluated the efficacy of gTME in *E. coli* by screening *rpoD* mutant library under cyclohexane pressure. We aimed at isolating σ^70^ mutants to improve the solvent tolerance of *E. coli*, which could potentially be applied in non-aqueous biocatalysis and biofuel production.

## Results

### Screening of solvent tolerance σ^70^ mutants

**Fig. 1:**
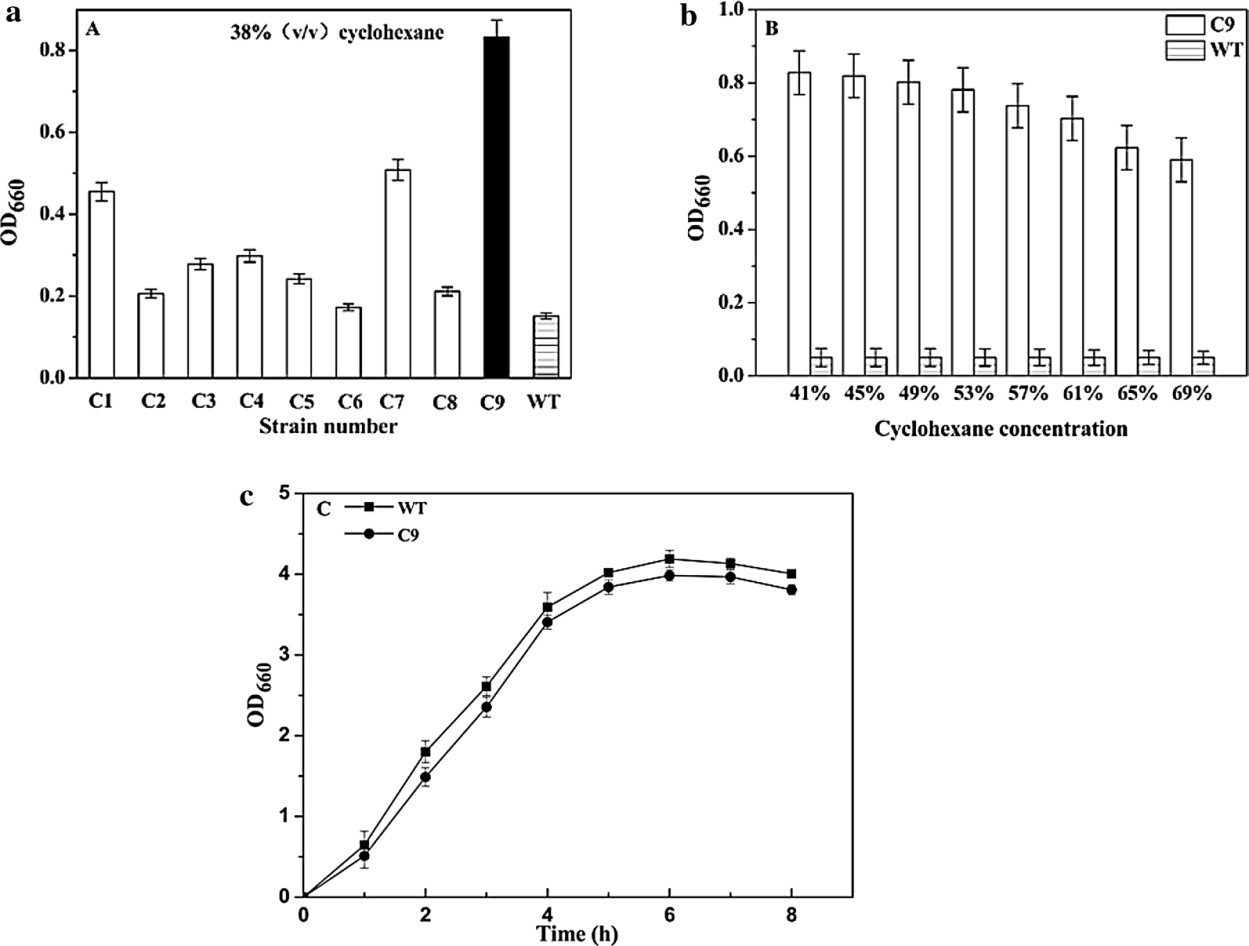
**a** Screening of solvent tolerance σ^70^ mutants C1–C9 at 38 % cyclohexane; **b** cyclohexane tolerance of *E. coli* JM109 harboring σ^70^ mutants C9 and WT; **c** cell growth of *E. coli* JM109 harboring C9 mutant and WT in absence of organic solvent. Strains were cultured at 37°C. For **a** and **b**, different concentration of cyclohexane was added when OD_660_ reached 0.2. Cell density was determined after 8 h of growth

To improve the solvent tolerance of *E. coli*, a *rpoD* mutant library was constructed and screened under cyclohexane pressure. After preliminary screening, 9 strains resulted in OD_660_ of over 1.1 were subjected to secondary screening, where mutants were enriched through repeated subcultures supplemented with escalating cyclohexane concentration. Finally, *E. coli* strain carrying σ^70^ mutant C9 showed the highest cyclohexane tolerance was selected. When grew in 38 % cyclohexane, its OD_660_ could reach 0.83, while the parent strain JM109/pHACM-*rpoD*^WT^ could not even grow under this condition (Fig. 1a). Then solvent tolerance of σ^70^ mutant C9 was determined under higher cyclohexane concentrations. The result showed that *E. coli* harboring C9 could tolerate 69 % cyclohexane (Fig. 1b). In the absence of cyclohexane, there was no significant difference between the cell growth of WT and C9 mutant strains (Fig. 1c), suggesting *rpoD* mutagenesis would not affect the normal growth of the *E. coli* strain.

**Fig. 2:**
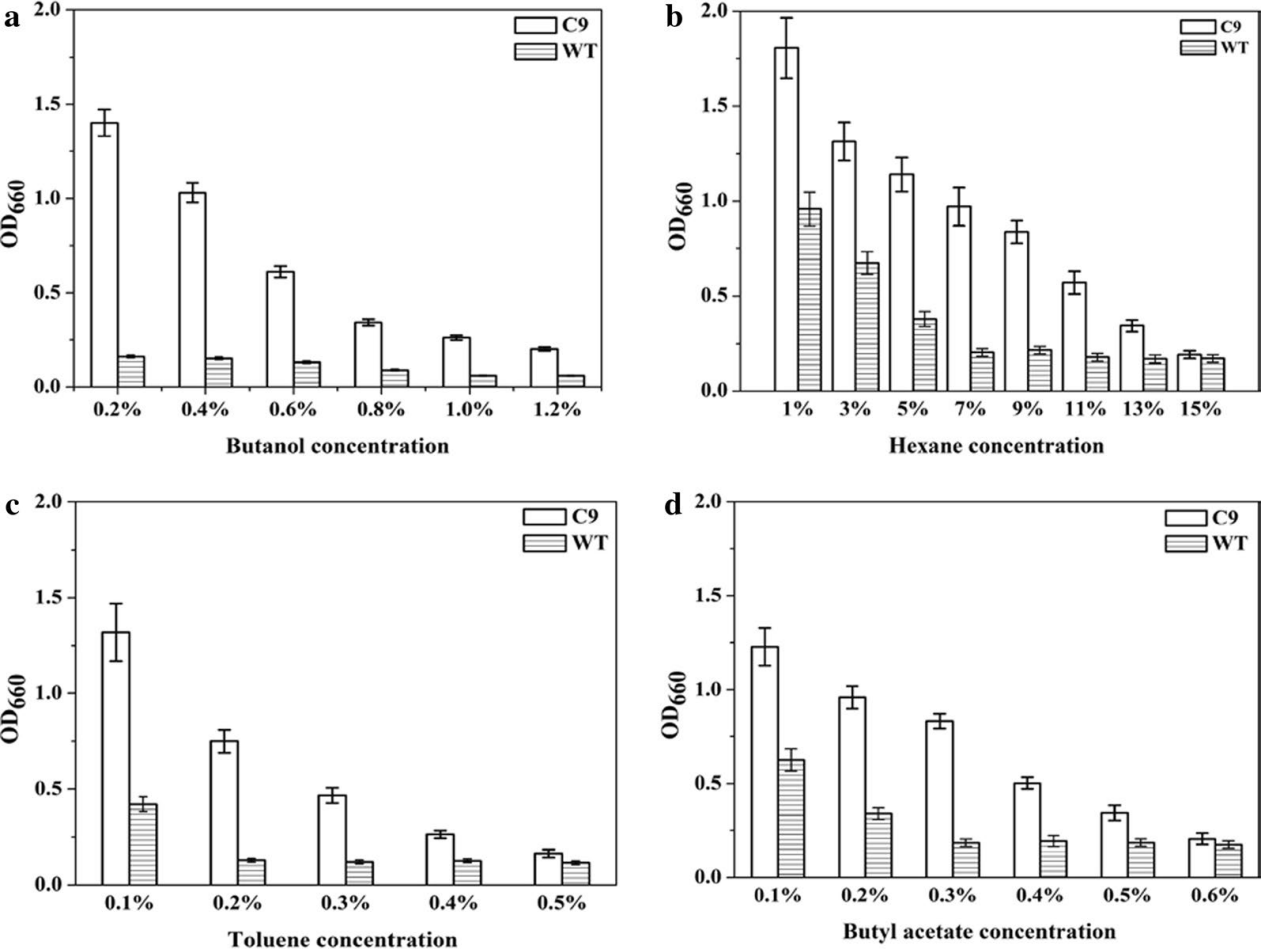
Solvent tolerance of *E. coli* JM109 harboring σ^70^ mutant C9 and WT towards different concentrations of butanol (**a**), hexane (**b**), toluene (**c**), and butyl acetate (**d**) Strains JM109/pHACM-*rpoD* ^C9^ and JM109/pHACM-*rpoD* ^WT^ were cultured at 37 °C. Different concentrations of butanol, hexane, toluene and butyl acetate were added when OD_660_ reached 0.2. Cell density was determined after 8 h of growth

Solvent tolerance towards other solvents was also investigated. *E. coli* carrying C9 showed increasing in cell density when cultivated in presence of 1.0 % butanol, 13 % hexane, 0.4 % toluene, and 0.5 % butyl acetate, whereas WT σ^70^ could merely survive under 0.1 % butanol, 5 % hexane, 0.1 % toluene and 0.2 % butyl acetate (Fig. 2).

Among various organic solvents, C9 mutant strain showed higher tolerance to cyclohexane. Since cyclohexane pressure was used in the library screening, isolated mutants often show higher preference to cyclohexane. Additionally, it has been proved that cyclohexane could be oxidized into cyclohexanol with less toxicity by microorganisms in our previous study [23].

The sequence alignment of σ^70^ WT and C9 revealed that *rpoD* mutant gene C9 contains two amino-acid mutations in region 1.1 (D39E, A72V) and two other mutations in region 1.2 (T94M, and a stop codon mutation at residue 123).

### 2-DE analysis and protein identification by MALDI-TOF/TOF

**Fig. 3:**
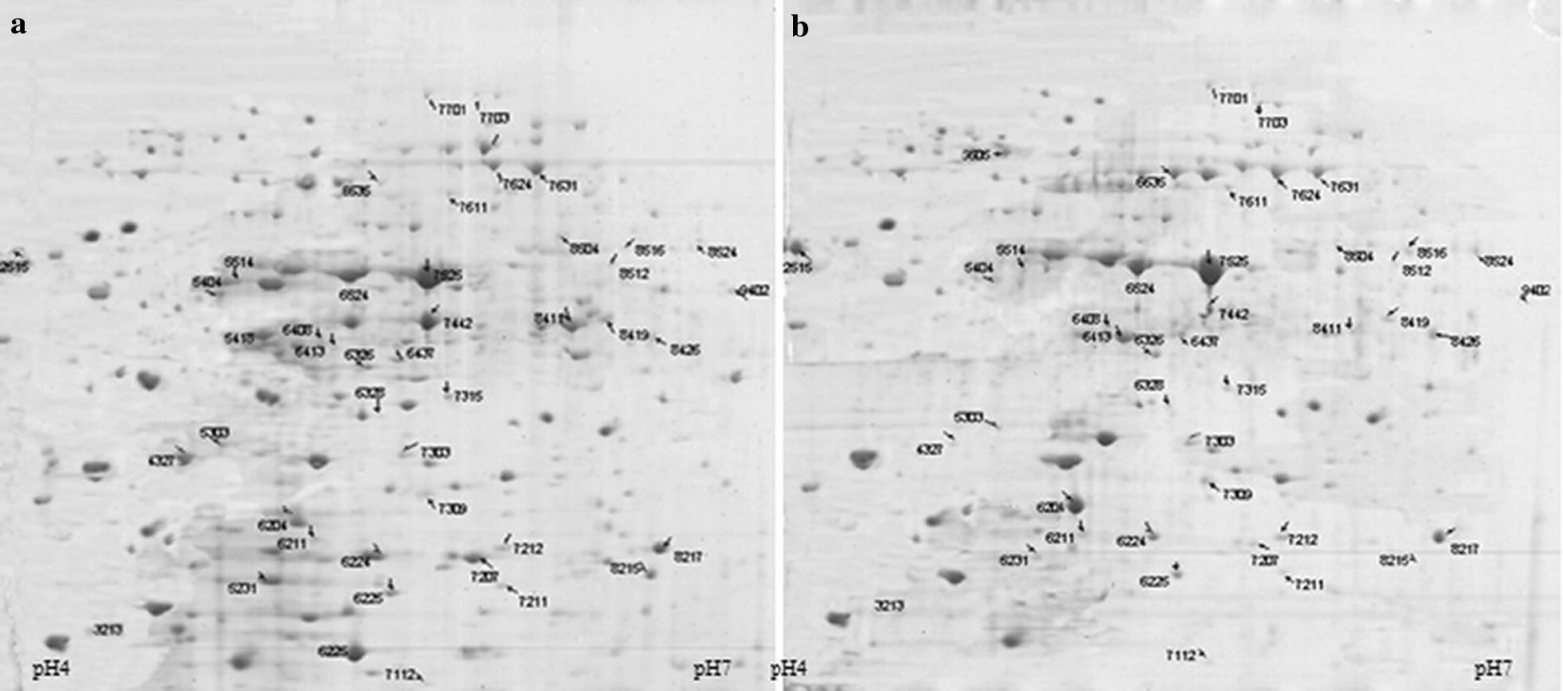
2D-PAGE of total proteins of *E. coli* JM109/pHACM-*rpoD* ^C9^ under different solvent treatments. **a** Without solvent; **b** with 38 % (v/v) cyclohexane. For each treatment condition, 2-DE experiment was conducted in triplicates

Two-DE, a powerful protein separation technique to illustrate proteins associated with certain phenotype, was used to investigate the proteomics of *E. coli* strains harboring C9 mutant when grown with or without cyclohexane. 2-DE analysis of WT strain (without cyclohexane) was also conducted as control. Our results show that there was no obvious difference between WT and C9 strain in the absence of cyclohexane (Additional file 1: Figure S1). Compared with control (C9 without solvent), 204 high-abundant proteins in C9 strain showed over twofold difference in the presence of 38 % cyclohexane (Fig. 3).

Among 204 high-abundant proteins, 43 independent protein spots were cut off and analyzed by MALDI-TOF/TOF. Finally, 22 proteins including 19 up-regulated and 3 down-regulated proteins were successfully identified (Table 1). These up-regulated proteins are involved in nucleotide synthesis, amino acid and glucose metabolism, transporter and porin proteins synthesis, etc.

**Table 1:** Proteins identification by MALDI-TOF/TOF

Spot no.	Homologous protein annotation	Coding gene	Mass (Da)	pI	Up/down regulation	Function
2512	Phosphotransferase enzyme family protein	UMNK88_2189	32,682	4.98	↑	Unknown
5231	Universal stress global response regulator	*uspA*	16,028	5.08	↑	Modulate and reorganize the carbon flow
5303	Conserved protein, UPF0070 family	*yfgM*	22,162	5.07	↑	Unknown
5514	Galactose-binding transport protein	*mglB*	35,720	5.68	↑	Galactose-binding transport
6204	Chain A, outer membrane protein	*ompX*	16,350	5.04	↑	Promote bacterial adhesion
6224	Global DNA-binding transcriptional dual regulator H-NS	*Hns*	15,331	5.24	↑	Global DNA-binding transcriptional dual regulator H-NS
6326	Chaperones protein HchA	*hchA*	31,190	5.63	↑	HSP31 molecular chaperone
6328	Chain A, outer membrane protein Ompw	*ompW*	21,661	6.03	↑	Acts as a receptor for colicin S4
6413	pspA Protein	*pspA*	25,562	5.51	↑	Regulatory protein for phage-shock-protein operon
6437	Adenylate kinase	*Adk*	23,712	6.01	↑	Energy metabolism
6524	Outer membrane protein OmpA	*ompA*	17,186	5.99	↑	Serves as a receptor for a number of T-even like phage
6635	Tryptophanase	*tnaA*	53,098	5.88	↑	Tryptophanase/l-cysteine desulfhydrase
7212	50S Ribosomal protein L9	*Prll*	15,759	6.15	↑	RNA-binding protein
7303	Superoxide dismutase	*soda*	15,974	5.95	↑	Destroys radicals in the cells
7315	ATP-dependent Clp protease, proteolytic subunit ClpP	*Clp*	23,286	5.52	↑	Acts to disaggregate proteins
8419	Two-component system phosphate regulon response regulator OmpR	*ompR*	26,757	6.01	↑	DNA-binding protein
7611	Aminopeptidase B	*pepB*	46,483	5.60	↑	Probably acts in intracellular peptide degradation
8425	Succinate dehydrogenase iron-sulfur subunit	*sdhB*	27,394	6.31	↑	Iron-sulfur protein subunit of succinate dehydrogenase
8524	Glyceraldehyde-3-phosphate dehydrogenase A	*gapA*	35,550	6.58	↑	Encodes one of the two subunits of GapA
5404	Outer membrane porin protein C	*ompC*	40,343	4.58	↓	Forms passive diffusion pores
4327	Thiol peroxidase	*Bcp*	17,995	5.03	↓	Bacterioferritin comigratory protein
7703	Chain A, dipeptide transporter	*dppA*	57,599	5.74	↓	Dipeptide-binding protein of a transport system that can be subject to osmotic shock

↑ Represents up-regulated genes; ↓ represents down-regulated genes

Among them, up-regulated genes *gapA* (glyceraldehyde-3-phosphate dehydrogenase A) and *sdhB* (FeS subunit of succinate dehydrogenase) are involved in the glycolysis process and TCA cycle, respectively [25, 26]. Both of them could produce intracellular ATP and provide high energy storage to overcome solvent stress. The expression levels of *pepB* (aminopeptidase B) and *yfgM* (a hypothetical protein) were remarkably enhanced in C9 mutant under solvent treatment, whereas their functions have barely been reported. Both *bcp* (thiol peroxidase) and *dppA* (dipeptide transporter) genes were significantly down-regulated. It has been reported that *bcp* is related to the organic solvent (such as phenol) and oxidative stress tolerance [27]. Gene *dppA* could function as dipeptide transporter, which might responsible for the organic solvent transportation into cytoplasm [28]. Therefore, these six genes (*gapA*, *sdhB*, *pepB*, *yfgM*, *dppA* and *bcp*) were selected for further characterization. Real-time RT-qPCR analysis confirmed that the transcription levels of 4 up-regulated genes (*yfgM*, *gapA*, *sdhB*, *pepB*) in C9 strain were enhanced for 15.6, 2.9, 4.2, and 12.4-fold under solvent treatment, and the transcription levels of 2 down-regulated genes (*bcp* and *dppA*) were decreased by 14.1 and 10 folds (Additional file 2: Figure S2).

### OST-related properties of candidate genes

#### SDS-PAGE analysis of retro-complementation strains

We constructed four gene deletion *E. coli* strains, JM109(Δ*yfgM*), JM109(Δ*sdhB*), JM109(Δ*gapA*) and JM109(Δ*pepB*), and their corresponding retro-complementary strains. The successful expression of these retro-complementary genes was confirmed by SDS-PAGE (Additional file 3: Figure S3). The molecular weights of YFGM, FeS subunit of succinate dehydrogenase, glyceraldehyde-3-phosphate dehydrogenase A, and Aminopeptidase B were estimated to be 22, 26, 35 and 46 kDa, respectively, in agreement with their theoretical values.

#### OST assay of up- and down-regulated genes

**Fig. 4:**
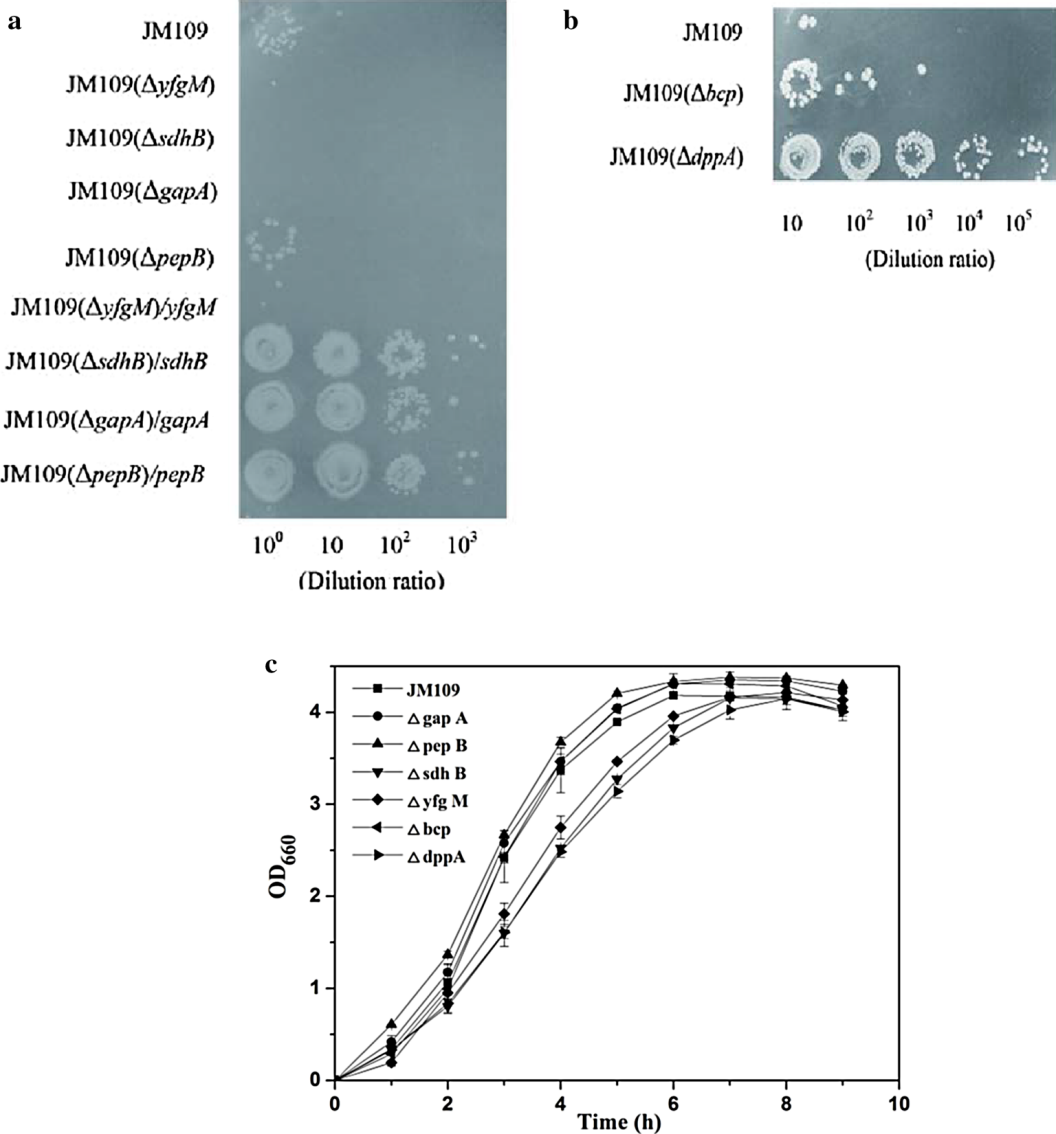
Colony formation efficiency of various *E. coli* knockout and retro-complementary strains of **a** up-regulated genes and **b** down-regulated genes under 1 % (v/v) cyclohexane; **c** cell growth of knockout strains in absence of cyclohexane. The cultures were spotted in 10-fold dilutions and incubated at 37 °C for 24 h

Colony-formation efficiency method was employed for OST assay. *E. coli* knockout strains were cultured in the presence of 1 % (v/v) cyclohexane. As shown in Fig. 4a, all four single-gene deletion strains, as well as retro-complementary strain JM109(Δ*yfgM*)/*yfgM* showed no tolerance to cyclohexane. For the other 3 retro-complementary strains, JM109(Δ*sdhB*)/*sdhB*, JM109(Δ*gapA*)/*gapA* and JM109(Δ*pepB*)/*pepB*, the colony-formation efficiency was over 10^3^ magnitude higher than that of the control (*E. coli* JM109), exhibiting remarkably higher cyclohexane tolerance than the control.

OST assay of down-regulated genes (*bcp* and *dppA*) was also performed. After overnight incubation, the colony-formation efficiency of JM109(Δ*dppA*) was over 10^4^ magnitude higher than that of the control (Fig. 4b). Strain JM109(Δ*bcp*) also showed slightly increased solvent tolerance.

To further evaluate the effect of gene deletion on cell growth under standard condition, six knockout strains were cultured in LB liquid medium without solvent. Figure 4c shows there was no obvious difference between the cell growth of JM109 and the knockout strains in the absence of cyclohexane. Similar cell density (OD_660_ around 4.2) was reached by all the strains after 8 h of growth, indicating these gene deletions would not affect the normal cell growth of *E. coli*.

### Effect of organic solvents on the intracellular ATP

It has been reported that the presence of organic solvent could lower intracellular ATP level [29]. When JM109 harboring σ^70^ C9 and WT were cultured without cyclohexane, the intracellular ATP concentrations were determined to be 2.65 ± 0.58 μmol/g and 2.98 ± 0.65 μmol/g. The presence of 1 % cyclohexane caused a decrease in ATP to 1.45 ± 0.46 μmol/g and 0.78 ± 0.34 μmol/g for C9 and WT, respectively (Additional file 4: Table S1). And while C9 was grown in LB with 38 % cyclohexane, the content of ATP was 0.56 μmol/g (data not shown). These results indicate that σ^70^ mutant C9 could help to maintain higher intracellular ATP level than its WT.

### Application of pHACM-*rpoD*^C9^ in whole-cell biotransformation

Using whole-cell biocatalyst *E. coli* BL21(DE3)/*Bm*GDH-*Cg*CR/pHACM^C9^, biocatalytic preparation of ethyl (*R*)-4-chloro-3-hydroxybutanoate [(*R*)-CHBE] catalyzed in aqueous/butyl acetate and aqueous/toluene biphasic reaction systems was attempted. After 2 h of reaction at 30 °C, (*R*)-CHBE was achieved in higher yields in both biphasic systems compared with control strain (without pHACM^C9^). At substrate (COBE) concentration of 100 g/L, optical pure (*R*)-CHBE was produced in 100 % yield in aqueous/butyl acetate biphasic system, and a bit lower yield of 94.6 % in aqueous/toluene system. Whereas lower yields of 79.5 and 85.5 % were obtained by the control strain in the same biphasic systems (Table 2).

**Table 2:** Asymmetric synthesis of (*R*)-CHBE in aqueous/organic solvent (1:1) biphasic systems

Strains	Enzyme activity (U/mg)		Yield (%)		*ee* (%)
	GDH	*Cg*CR	Aqueous/butyl acetate	Aqueous/toluene	
*E. coli*/BmGDH-*Cg*CR	9.5	0.9	79.5	85.5	>99
*E. coli*/BmGDH-*Cg*CR/pHACM^C9^	8.9	0.8	100	94.6	>99

Reaction conditions: 0.5 g wet cells, 250 g/L glucose, 100 g/L of ethyl 4-chloro-3-oxobutanoate (COBE) in 5 mL potassium phosphate buffer (0.1 mol/L, pH 6.5) and 5 mL organic solvent, in 30 °C shaker for 2 h

## Discussion

The organic-solvent resistance of microorganisms is important for their applications in non-aqueous whole-cell biocatalysis and biofuel (such as alcohol) fermentation processes. In our previous study, an OST strain (*P. putida* JUCT1), capable of growing in 60 % (v/v) cyclohexane, was isolated following gradient solvent adaptation [23]. Singh and co-workers developed an OST-adapted strain *P. putida* which could grow in the presence of 30 % (v/v) cyclohexane [30]. In recent years, global transcriptional engineering (gTME) approach has been successfully utilized to improve the stress-resistance performance of microbial cells [15]. Zhang and co-workers also reported the isolation of *E. coli* strain tolerating 1.2 % (v/v) butanol using this approach [9]. Here, gTME was adopted to enhance the solvent tolerance of *E. coli*. After two rounds of screening, an OST *E. coli* strain harboring σ^70^ could survive in the presence of 69 % cyclohexane. Furthermore, C9 mutant did not affect the normal growth of *E. coli* strains.

OST assay indicates that four genes (*gapA*, *sdhB,pepB* and *dppA*) play critical roles in OST of *E. coli*. Glyceraldehyde-3-phosphate dehydrogenase A (*gapA*) is involved in glycolysis process that could produce pyruvic acid and ATP [25]. FeS subunit of succinate dehydrogenase (*sdhB*) is involved in TCA cycle and could provide coenzyme for succinate dehydrogenase that catalyzes the synthesis of fumarate and ATP [26]. Consequently, up-regulated expression of *gapA* and *sdhB* can increase the intracellular ATP level, providing high energy storage. Organic solvents usually have negative effect on the intracellular ATP level. On one hand, most of organic solvents could partially inhibit the membrane ATPase and lowered the intracellular ATP level [29]. On the other hand, cells need to consume more ATP to provide energy to pump organic solvents out of the cells and decrease its toxicity [31]. Therefore, mutant C9 with high intracellular ATP level will result in improved OST for cells.

The solvent-tolerant mechanism of *pepB* is still unknown and needs to be further studied. The *dppA* knockout could improve solvent tolerance of *E. coli*, since *dppA* could function as dipeptide transporter that was reported to be responsible for organic solvent transportation into cytoplasm [28]. However, retro-complementation of *yfgM*, an up-regulated gene, did not improve the solvent tolerance of *E. coli* significantly. It is speculated that synergistic effect also plays critical roles in the OST-related functions of a number of genes, such as *yfgM*. In other studies, a number of critical genes involved in solvent tolerance phenotype of microorganisms had been identified. For example, Honda’s group reported that the solvent tolerance of *E. coli* was markedly enhanced by overexpression of *manXYZ* [32] and *purR* [33]. Some OST-related genes were listed (Additional file 5: Table S2).

**Fig. 5:**
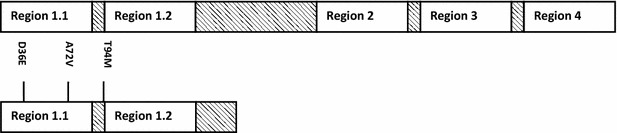
Genetic composition of σ^70^ WT and C9

There are 7 σ factors (σ^70^, σ^54^, σ^32^, σ^S^, σ^E^, σ^F^ and σ^FecI^) in *E. coli*, and σ^70^ is the main control factor that is responsible for the transcription of over 1000 genes. σ^70^ consists of 4 parts, namely σ^1^ (region 1.1), σ^2^ (region 1.2 and region 2), σ^3^ (region 3) and σ^4^ (region 4). In 2007, Alper and Stephanopoulos generated σ^70^ mutant gene which retained only σ^4^ region to enhance the ethanol tolerance of *E. coli* [21]. In 2008, Yu and coworkers isolated σ^70^ mutant comprised of only σ^1^ and σ^2^ for enhanced hyaluronic acid accumulation [22]. In this study, a truncated mutation also occurred in *rpoD* mutant C9, remaining regions σ^1.1^and σ^1.2^ (Fig. 5). Our results indicate that a truncated mutation in *rpoD* may markedly change cell phenotype such as OST by regulating the transcription of a number of related genes.

Zhang and coworkers reported that cell morphological may change to adapt to the harsh condition, such as solvent environment [9]. In this study, both *E. coli* strains harboring WT and C9 σ^70^ exhibited a much elongated and narrower shape in the presence of 4.0 % (v/v) cyclohexane, compared with those without solvent (data not shown). It is presumed that a lower specific surface area of *E. coli* harboring C9 could conduce to its higher solvent tolerance.

In this study, the potential of σ^70^ mutant in whole-cell biocatalysis in aqueous/solvent biphasic systems was also validated. Using a recombinant *E. coli* strain transformed with pHACM^C9^, higher yield was achieved in different biphasic reaction systems containing extremely toxic solvents, i.e. butyl acetate (Log*P* = 1.7) and toluene (Log*P* = 2.5). It suggests that σ^70^ mutant could conduce to enhanced whole-cell biocatalytic efficiency in non-aqueous system by enhancing solvent tolerance of microbial cells.

## Conclusions

Microbial OST mechanisms are complicated and regulated by multi-mechanisms. In this study, random mutagenesis of σ^70^ is a feasible and efficient approach for engineering strain with OST-phenotype. Based on proteomic analysis, several genes (*gapA, sdhB, pepB* and *dppA*) contributed to the enhanced solvent tolerance of *E. coli*. Additionally, our results provide molecular basis to construct OST strains for industrial applications such as biofuel production and non-aqueous biocatalysis.

## Methods

### Bacterial strains and chemicals

Organic-solvent sensitive *E. coli* JM109 was used as the starting strain and was cultured as previously described [24]. Strains and plasmids used in this study are shown in Table 3. Primers used in this study are listed (Additional file 6: Table S3). PrimeSTAR^®^HS DNA Polymerase and restriction enzymes were purchased from Takara (Tokyo, Japan). GeneMorph II Random Mutagenesis Kit was purchased from Stratagene (La Jolla, CA, USA). The dam-methylated DNA specific restriction enzyme *Dpn*I was purchased from New England Biolabs (Ipswich, MA, USA). Cyclohexane, toluene, and other solvents were obtained from Sinopharm Chemical Reagent (Shanghai, China).

**Table 3:** Strains and plasmids used in this study

Strains and plasmids	Genotype	Reference
*Escherichia coli* strains		
JM109	F’(*tra*D36, *pro*AB^+^, *lac*I^q^, Δ(*lac*Z)M15) *end*A1 *rec*A1 *hsd*R17 (r^−^ K, m^+^ K) *mcr*A *sup*E44 λ^−^ *gyr*A96 *rel*A1 Δ(*lac*-*pro*AB) *thi* ^−1^	Takara
JM109(ΔyfgM)	Same as JM109, but with Δ*yfg*M	This study
JM109(Δ*sdhB*)	Same as JM109, but with Δ*sdh*B	This study
JM109(Δ*gapA*)	Same as JM109, but with Δ*gap*A	This study
JM109(Δ*pepB*)	Same as JM109, but with Δ*pep*B	This study
JM109(Δ*bcp*)	Same as JM109, but with Δ*bcp*	This study
JM109(Δ*dppA*)	Same as JM109, but with Δ*dpp*A	This study
JM109(Δ*yfgM*)/*yfgM*	Same as JM109, but with Δ*yfg*M/pQE-*yfg*M	This study
JM109(Δ*sdhB*)/*sdhB*	Same as JM109, but with Δ*sdh*B/pQE-*sdh*B	This study
JM109(Δ*gapA*)/*gapA*	Same as JM109, but with Δ*gapA*/gapA	This study
JM109(Δ*pepB*)/*pepB*	Same as JM109, but with Δ*pep*B*/*pQE-*pep*B	This study
Plasmids		
pKD13, pKD46, and pCP20	Expression vector	34
pQE80L	Expression vector	QIAGEN
pHACM-*rpoD* ^WT^	Plasmid harboring WT *rpoD*	22
pHACM-*rpoD* ^C9^	Plasmid harboring *rpoD* mutant C9	This study

### Construction of random mutagenesis library

Random mutagenesis of *rpoD* was performed with GeneMorph^®^ II Random Mutagenesis Kit using a low-copy number plasmid pHACM-*rpoD*^WT^ (a kind gift from Dr. Huimin Yu, Tsinghua University) as template (around 120 ng, aiming at 5–9 mutations/kb) [22]. Whole plasmid PCR was performed to accomplish library construction, and the amplified recombinant plasmids were transformed into *E. coli* JM109 after digestion by *Dpn*I. *E. coli* transformants were spread on LB agar plate containing 34 μg/mL of chloramphenicol, and the colonies were scraped off to form *rpoD* mutant library for further OST phenotype selection. The total library size of *rpoD* mutants was approximately 10^6^.

### Phenotype selection

#### Primary screening

The *rpoD* mutant library was inoculated into LB/Cm liquid medium. When OD_660_ reached 0.2, 4 % (v/v) cyclohexane was added into the culture. After 8 h of growth at 200 rpm and 37 °C, cells were spread onto LB/Cm agar plates. Colonies from agar plate were picked and inoculated into LB/Cm liquid medium supplemented with 4 % (v/v) cyclohexane. JM109/pHACM-*rpoD*^WT^ was used as the control. After 8 h of incubation at 200 rpm and 37 °C, cell density was measured and 9 mutants with OD_660_ above 1.1 were chosen for re-screening.

#### Secondary screening

Better mutants selected were further cultured under higher cyclohexane (4–70 %) concentrations by 2 % (v/v) gradient. The cell density was measured after 8 h of growth. Finally, the best mutant named C9 was chosen.

The solvents were supplemented in volume percentage unless otherwise stated.

### Resistance to various organic solvents

*Escherichia coli* strain harboring σ^70^ mutant C9 was incubated at 37 °C and 220 rpm for overnight, and the overnight culture was inoculated (1.0 %, v/v) into fresh medium. Different concentrations of butanol, hexane, toluene and butyl acetate were added when OD_660_ reached 0.2. Cell density was analyzed by measuring OD_660_ after incubation for 8 h.

### Extraction of total cellular protein

*Escherichia coli* strain harboring σ^70^ mutant C9 was cultured overnight without (as control) or with 38 % cyclohexane. Cells were harvested by centrifugation at 4000×*g* and 4 °C for 10 min, and washed three times with cold deionized water. Then cell pellets were resuspended in lysis buffer (2 M thiourea, 8 M urea, 65 mM dithiothreitol, 40 mM Tris-base, 4 % (w/v) CHAPS, and 0.001 % (w/v) bromophenol blue), and treated with ultrasonication (300 W, pulse 1 s, pause 3 s for 15 min) in ice water bath. The total cellular protein was obtained by centrifugation at 15,000×*g* and 4 °C for 15 min to remove the cell debris. Protein concentration was measured using Sangon Biotech Non-Interference Protein Assay Kit (Shanghai, China). All protein samples were stored at −80 °C for further 2-D electrophoresis analysis.

### 2-D electrophoresis and protein identification by MALDI-TOF/TOF

2-D electrophoresis was performed as previous described [22]. Briefly, the total cellular protein samples (800 μg per sample) were subjected to 2-DE on IPG pH 4–7 strips (24 cm, GE Healthcare). In order to obtain wide distribution of all proteins spots, IPG 3–10 strips (7 cm, GE Healthcare, Pittsburgh, PA, USA) was initially used in isoelectric focusing electrophoresis (IEF). The result indicates that most proteins were located over pH range of 4–7. Therefore, IPG 4–7 strips (24 cm, GE Healthcare) were chosen. The isoelectric focusing was performed at 20 °C using the following program: 150 V for 1 h, gradient to 250 V for 1 h, gradient to 500 V for 1.5 h, gradient to 1000 V for 2.5 h, gradient to 5000 V for 4 h, gradient to 10,000 V for 2.5 h, holding at 10,000 V, 90,000 V/h, and for the total of 65,000 V/h. Then, each strip was equilibrated, washed twice and transferred onto 12 % SDS-polyacrylamide gels. After 2-DE, the destained gel images obtained by ImageScanner III (GE Healthcare, PA, USA) was analyzed by PDQuest™2-D Analysis Software (Bio-Rad, CA, USA). For each treatment condition, 2-DE experiment was conducted in triplicate.

Protein spots were identified by MALDI-TOF/TOF using the method described previously [22].

### RNA isolation and real-time quantitative reverse transcription PCR

Total cellular RNA was extracted from *E. coli* strain harboring σ^70^ mutant C9 grown overnight with or without 38.0 % (v/v) cyclohexane using the Simply P Total RNA Extraction Kit (BioFlux, Japan). Reverse transcription step was carried out using RevertAid First Strand cDNA Synthesis Kit (Thermo, USA) with random primer mix following the manufacturer’s manual. Real-time quantitative reverse transcription PCR (RT-qPCR) was performed with RealMasterMix (SYBR Green) (TIANGEN, China) using Bio-Rad iQ5 real-time PCR detection system (Bio-Rad, USA). The bacterial 16S rRNA gene sequence was used as a reference gene in real-time PCR (Additional file 6: Table S3). The real-time PCR conditions were as follows: 1 min at 94 °C, 35 cycles at 94 °C for 10 s, followed by 55 °C for 30 s and 68 °C for 15 s. To analyze the gene expression level, ΔΔCt method was chosen and the standard curves of each primer were plotted to ensure similar amplification efficiency compared with the reference gene.

### Gene knockout and retro-complementation

Using Red-mediated recombination [34], six genes including four up-regulated (*gapA, sdhB, pepB, yfgM)* and two down-regulated *(dppA* and *bcp*) genes were knocked out from genome of *E. coli* JM109 (Table 3). Meanwhile, retro-complementation strains of four up-regulated genes (*gapA, sdhB, pepB* and *yfgM*) were also generated using pQE80L as expression vector. The expression of these genes was analyzed by SDS-PAGE after induction with 0.2 mM IPTG.

### Colony formation efficiency assay

For four up-regulated genes (*gapA*, *sdhB*, *pepB* and *yfgM*) and two down-regulated genes (*dppA* and *bcp*), six knockout strains and four corresponding retro-complementation strains of up-regulated genes were cultured in LB medium. Strains JM109 was used as control. For four retro-complementation strains, 0.2 mM IPTG were added when OD_660_ reached 0.2 to initiate induction. For all strains, 1 % cyclohexane was added when OD_660_ reached 1.0. The cells were further cultivated for 90 min. Then the cultures were diluted for 10^5^, 10^4^, 10^3^ and 10^2^, 10 folds with aseptic water. Then 10 μL of the diluted culture was spread onto LB ager plate, and further incubated at 37 °C for 20 h.

### Quantification of intracellular ATP

For *E. coli* strains harboring σ^70^ WT or C9, cyclohexane [1 % (v/v)] was added at exponential phase of. Cells were harvested at stationary phases by centrifugation at 4000×*g* and 4 °C for 10 min. The cells were treated with lysis solution, and ATP concentrations were determined with ATP bioluminescence assay kit from Beyotime (Haimen, China) based on fluorescence intensity. The intracellular ATP content was calculated according to the standard curve.

### Whole-cell biocatalysis in biphasic systems

Plasmid pHACM-*rpoD*^C9^ was transformed into *E. coli* BL21(DE3)*/Bm*GDH-*Cg*CR harboring a carbonyl reductase and glucose dehydrogenase, which had been previously constructed for the asymmetric synthesis of ethyl (*R*)-4-chloro-3-hydroxybutanoate [(*R*)-CHBE], a chiral intermediate for the synthesis of l-carnitine [35]. The resulted strain *E. coli* BL21(DE3)/*Bm*GDH-*Cg*CR/pHACM^C9^ was applied as whole-cell biocatalyst in aqueous/solvent biphasic systems. And *E. coli* BL21(DE3)*/Bm*GDH-*Cg*CR was used as control. Reaction mixture consists of 0.5 g wet cells, 250 g/L glucose, 100 g/L of ethyl 4-chloro-3-oxobutanoate (COBE) in 5 mL potassium phosphate buffer (0.1 mol/L, pH 6.5) and 5 mL organic solvent, and was incubated in a 30 °C shaker for 2 h. The reaction mixture was extracted by ethyl acetate for further GC analysis.

## Additional files

10.1186/s12934-015-0368-4 2D-PAGE of total proteins of *E. coli* JM109/pHACM-*rpoD* ^WT^ without solvent.
10.1186/s12934-015-0368-4 Real-time qRT-PCR of up- and down-regulated genes.
10.1186/s12934-015-0368-4 Recombinant expression of *pepB, gapA, sdhB* and *yfgM* in *E. coli* JM109 and their corresponding knockout strains.
10.1186/s12934-015-0368-4 Intracellular ATP concentration in *E. coli* harboring WT and C9 mutant *rpoD*.
10.1186/s12934-015-0368-4 OST-related genes identified in microorganisms.
10.1186/s12934-015-0368-4 Primers used in this study.

## References

[1] Luque R, Herrero-Davila L, Campelo JM, Clark JH, Hidalgo JM, Luna D, Marinas JM, Romero AA (2008). Biofuels: a technological perspective. Energy Environ Sci.

[2] de Carvalho CC (2011). Enzymatic and whole cell catalysis: finding new strategies for old processes. Biotechnol Adv.

[3] Heipieper HJ, Neumann G, Cornelissen S, Meinhardt F (2007). Solvent-tolerant bacteria for biotransformations in two-phase fermentation systems. Appl Microbiol Biotechnol.

[4] Inoue A, Horikoshi K (1991). Estimation of solvent-tolerance of bacteria by the solvent parameter log P. J Ferment Bioeng..

[5] Sardessai Y, Bhosle S (2002). Tolerance of bacteria to organic solvents. Res Microbiol.

[6] Inoue A, Horikoshi K (1989). A Pseudomonas thrives in high concentrations of toluene. Nature.

[7] Mi J, Becher D, Lubuta P, Dany S, Tusch K, Schewe H, Buchhaupt M, Schrader J (2014). De novo production of the monoterpenoid geranic acid by metabolically engineered *Pseudomonas putida*. Microb Cell Fact.

[8] Alsaker KV, Paredes C, Papoutsakis ET (2010). Metabolite stress and tolerance in the production of biofuels and chemicals: gene-expression-based systems analysis of butanol, butyrate, and acetate stresses in the anaerobe *Clostridium acetobutylicum*. Biotechnol Bioeng.

[9] Zhang HF, Chong HQ, Ching CB, Song H, Jiang RR (2012). Engineering global transcription factor cyclic AMP receptor protein of *Escherichia coli* for improved 1-butanol tolerance. Appl Microbiol Biotechnol.

[10] Tsukagoshi N, Aono R (2000). Entry into and release of solvents by *Escherichia coli* in an organic-aqueous two-liquid-phase system and substrate specificity of the AcrAB-TolC solvent-extruding pump. J Bacteriol.

[11] Rojas A, Segura A, Guazzaroni ME, Teran W, Hurtado A, Gallegos MT, Ramos JL (2003). In vivo and in vitro evidence that TtgV is the specific regulator of the TtgGHI multidrug and solvent efflux pump of *Pseudomonas putida*. J Bacteriol.

[12] Minty JJ, Lesnefsky AA, Lin FM, Chen Y, Zaroff TA, Veloso AB, Xie B, McConnell CA, Ward RJ, Schwartz DR, Rouillard JM, Gao Y, Gulari E, Lin XN (2011). Evolution combined with genomic study elucidates genetic bases of isobutanol tolerance in *Escherichia coli*. Microb Cell Fact.

[13] Lin YL, Blaschek HP (1983). Butanol production by a butanol-tolerant strain of *Clostridium acetobutylicum* in extruded corn broth. Appl Environ Microbiol.

[14] Hermann M, Fayolle F, Marchal R, Podvin L, Sebald M, Vandecasteele JP (1985). Isolation and characterization of butanol-resistant mutants of *Clostridium acetobutylicum*. Appl Environ Microbiol.

[15] Alper H, Moxley J, Nevoigt E, Fink GR, Stephanopoulos G (2006). Engineering yeast transcription machinery for improved ethanol tolerance and production. Science.

[16] Klein-Marcuschamer D, Stephanopoulos G (2008). Assessing the potential of mutational strategies to elicit new phenotypes in industrial strains. Proc Natl Acad Sci USA.

[17] Park KS, Lee DK, Lee H, Lee Y, Jang YS, Kim YH, Yang HY, Lee SI, Seol W, Kim JS (2003). Phenotypic alteration of eukaryotic cells using randomized libraries of artificial transcription factors. Nat Biotechnol.

[18] Liu HM, Yan M, Lai CG, Xu L, Ouyang PK (2010). gTME for improved xylose fermentation of *Saccharomyces cerevisiae*. Appl Biochem Biotechnol.

[19] Siegele DA, Hu JC, Walter WA, Gross CA (1989). Altered promoter recognition by mutant forms of the σ 70 subunit of *Escherichia coli* RNA polymerase. J Mol Biol.

[20] Gardella T, Moyle H, Susskind MM (1989). A mutant *Escherichia coli* sigma 70 subunit of RNA polymerase with altered promoter specificity. J Mol Biol.

[21] Alper H, Stephanopoulos G (2007). Global transcription machinery engineering: a new approach for improving cellular phenotype. Metabolic Eng.

[22] Yu H, Tyo K, Stephanopoulos G (2008). A high-throughput screen for hyaluronic acid accumulation in recombinant *Escherichia coli* transformed by libraries of engineered sigma factors. Biotechnol Bioeng.

[23] Ni Y, Song L, Qian XH, Sun ZH (2013). Proteomic analysis of *Pseudomonas putida* reveals an organic solvent tolerance-related gene mmsB. PLoS One.

[24] Qian XH, Song L, Ni Y (2014). Enhanced organic solvent tolerance of *Escherichia coli* by 3-hydroxyacid dehydrogenase family genes. Appl Biochem Biotechnol.

[25] Charpentier B, Branlant C (1994). The *Escherichia coli* gapA gene is transcribed by the vegetative RNA polymerase holoenzyme E sigma 70 and by the heat shock RNA polymerase E sigma 32. J Bacteriol.

[26] Cooley JW, Howitt CA, Vermaas WFJ (2000). Succinate: quinol oxidoreductases in the cyanobacterium *Synechocystis* sp. strain PCC 6803: presence and function in metabolism and electron transport. J Bacteriol.

[27] Santos PM, Benndorf D, Sá-Correia I (2004). Insights into *Pseudomonas putida* KT2440 response to phenol-induced stress by quantitative proteomics. Proteomics.

[28] Kang A, Chang MW (2012). Identification and reconstitution of genetic regulatory networks for improved microbial tolerance to isooctane. Mol Biosyst.

[29] Bowles LK, Ellefson WL (1985). Effects of butanol on *Clostridium acetobutylicum*. Appl Environ Microbiol.

[30] Singh SK, Singh SK, Tripathi VR, Khare SK, Garg SK (2011). A novel psychrotrophic, solvent tolerant *Pseudomonas putida* SKG-1 and solvent stability of its psychro-thermoalkalistable protease. Process Biochem.

[31] Reyes LH, Almario MP, Kao KC (2011). Genomic library screens for genes involved in *n*-butanol tolerance in *Escherichia coli*. PLoS One.

[32] Okochi M, Kurimoto M, Shimizu K, Honda H (2007). Increase of organic solvent tolerance by overexpression of manXYZ in *Escherichia coli*. J Appl Microbiol Biotechnol.

[33] Shimizu K, Hayashi S, Doukyu N, Kobayashi T, Honda H (2005). Time-course data analysis of gene expression profiles reveals purR regulon concerns in organic solvent tolerance in *Escherichia coli*. J Biosci Bioeng.

[34] Datsenko KA, Wanner BL (2000). One-step inactivation of chromosomal genes in* Escherichia coli* K-12 using PCR products. Proc Natl Acad Sci USA.

[35] Xu GC, Yu HL, Zhang ZJ, Xu JH (2013). Stereocomplementary bioreduction of β-ketonitrile without ethylated byproduct. Org Lett..

