# Supplementary material for: Significantly improved solvent tolerance of Escherichia coli by global transcription machinery engineering
Source: Microb Cell Fact. 2015 Nov 5;14:175. doi: 10.1186/s12934-015-0368-4 (PMC4635540; doi:10.1186/s12934-015-0368-4)
Supplement: Supplementary file 3 — 10.1186/s12934-015-0368-4 Recombinant expression of pepB, gapA, sdhB and yfgM in E. coli JM109 and their corresponding knockout strains. [file 12934_2015_368_MOESM3_ESM.docx]

**Significantly improved solvent tolerance of *Escherichia coli* by global transcription machinery engineering**

**Additional files**

**Additional file 3**: Recombinant expression of *pepB, gapA, sdhB* and *yfgM* in *E. coli* JM109 and their corresponding knockout strains (Figure S3).


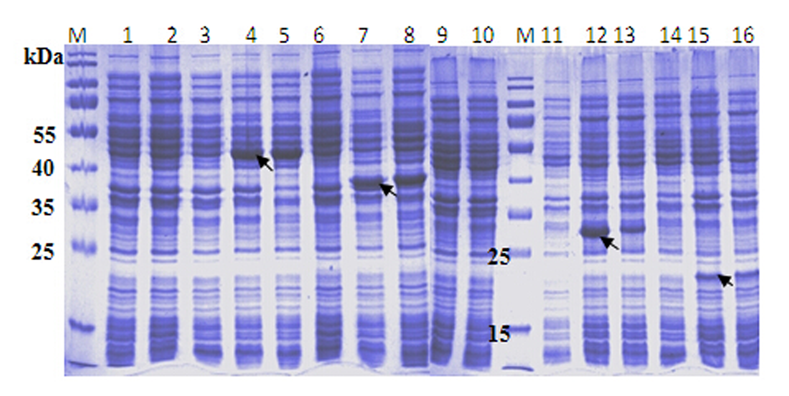


**Figure S3.** Recombinant expression of *pepB*, *gapA, sdhB* and *yfgM* in *E. coli* JM109 and their corresponding knockout strains.

Lane M: Marker; Lane 1: JM109; Lane 2: JM109/pQE80L; Lanes 3, 6, 9, 10, 11 and 14: whole-cell of JM109(Δ*pepB*), JM109(Δ*gapA*), JM109(Δ*bcp*), JM109(Δ*dppA*), JM109(Δ*sdhB*), JM109(Δ*yfgM*); Lanes 4 and 5: whole-cell and supernatant of JM109(Δ*pepB*)/*pepB*; Lanes 7 and 8: whole-cell and supernatant of JM109(ΔgapA)/gapA; Lanes 12 and 13: whole-cell and supernatant of JM109(Δ*sdhB*)/*sdhB*; Lanes 15 and 16: whole-cell and supernatant of JM109(Δ*yfgM*)/*yfgM*.

**References**

1. Ni Y, Song L (2013) Proteomic analysis of *Pseudomonas putida* reveals an organic solvent tolerance-related gene *mmsB*. PLoS ONE 8:e55858

2. Okochi M, Kurimoto M, Shimizu K, Honda H (2007) Increase of organic solvent tolerance by overexpression of *manXYZ* in *Escherichia coli*. Microbiol Biotechnol 73: 1394-1399

3. Nakajima H, Kobayashi K, Kobayashi M, Asako H, Aono R (1995) Overexpression of the *robA* gene increases organic solvent tolerance and multiple antibiotic and heavy metal ion resistance in *Escherichia coli*. Appl Environ Microbiol 61: 2302-2307

4. Hayashi S, Aono R, Hanai T, Mori H, Kobayashi T, Honda H (2003) Analysis of organic solvent tolerance in *Escherichia* coli using gene expression profiles from DNA microarrays. J Biosc Bioeng 95: 379-383

5. Basak S (2013) Improving *E. coli* performance under stress by rewiring its global regulator Camp Receptor Protein (CRP). Dissertation. Nanyang Technological University, Singapore
