# Supplementary material for: Significantly improved solvent tolerance of Escherichia coli by global transcription machinery engineering
Source: Microb Cell Fact. 2015 Nov 5;14:175. doi: 10.1186/s12934-015-0368-4 (PMC4635540; doi:10.1186/s12934-015-0368-4)
Supplement: Supplementary file 4 — 10.1186/s12934-015-0368-4 Intracellular ATP concentration in E. coli harboring WT and C9 mutant rpoD. [file 12934_2015_368_MOESM4_ESM.docx]

**Significantly improved solvent tolerance of *Escherichia coli* by global transcription machinery engineering**

**Additional files**

**Additional file 4**: Intracellular ATP concentration in *E. coli* harboring WT and C9 mutant *rpoD* (Table S1).

**Table S1** Intracellular ATP concentration in *E. coli* harboring WT and C9 mutant *rpoD* at exponential phase

| Strain | Intracellular ATP (μmol·g^−1^) | |
| --- | --- | --- |
|  | Without cyclohexane | 1% (v/v) cyclohexane |
| JM109/pHACM-*rpoD*^WT^ | 2.98 ± 0.65 | 0.78 ± 0.34 |
| JM109/pHACM-*rpoD*^C9^ | 2.65 ± 0.58 | 1.45 ± 0.46 |

**References**

1. Ni Y, Song L (2013) Proteomic analysis of *Pseudomonas putida* reveals an organic solvent tolerance-related gene *mmsB*. PLoS ONE 8:e55858

2. Okochi M, Kurimoto M, Shimizu K, Honda H (2007) Increase of organic solvent tolerance by overexpression of *manXYZ* in *Escherichia coli*. Microbiol Biotechnol 73: 1394-1399

3. Nakajima H, Kobayashi K, Kobayashi M, Asako H, Aono R (1995) Overexpression of the *robA* gene increases organic solvent tolerance and multiple antibiotic and heavy metal ion resistance in *Escherichia coli*. Appl Environ Microbiol 61: 2302-2307

4. Hayashi S, Aono R, Hanai T, Mori H, Kobayashi T, Honda H (2003) Analysis of organic solvent tolerance in *Escherichia* coli using gene expression profiles from DNA microarrays. J Biosc Bioeng 95: 379-383

5. Basak S (2013) Improving *E. coli* performance under stress by rewiring its global regulator Camp Receptor Protein (CRP). Dissertation. Nanyang Technological University, Singapore
