# Supplementary material for: Significantly improved solvent tolerance of Escherichia coli by global transcription machinery engineering
Source: Microb Cell Fact. 2015 Nov 5;14:175. doi: 10.1186/s12934-015-0368-4 (PMC4635540; doi:10.1186/s12934-015-0368-4)
Supplement: Supplementary file 5 — 10.1186/s12934-015-0368-4 OST-related genes identified in microorganisms. [file 12934_2015_368_MOESM5_ESM.docx]

**Significantly improved solvent tolerance of *Escherichia coli* by global transcription machinery engineering**

**Additional files**

**Additional file 5**: OST-related genes identified in microorganisms (Table S2).

**Table S2** OST-related genes identified in microorganisms

| Source | Gene | Reference |
| --- | --- | --- |
| *Pseudomonas putida* | *mmsB* | 1 |
| *E. coli* | *manXYZ* | 2 |
| *E. coli* | *robA* | 3 |
| *E. coli* | *marA* | 4 |
| *E. coli* | *crp* | 5 |
| *E. coli* | *gapA, sdhB, pepB* | This study |

**References**

1. Ni Y, Song L (2013) Proteomic analysis of *Pseudomonas putida* reveals an organic solvent tolerance-related gene *mmsB*. PLoS ONE 8:e55858

2. Okochi M, Kurimoto M, Shimizu K, Honda H (2007) Increase of organic solvent tolerance by overexpression of *manXYZ* in *Escherichia coli*. Microbiol Biotechnol 73: 1394-1399

3. Nakajima H, Kobayashi K, Kobayashi M, Asako H, Aono R (1995) Overexpression of the *robA* gene increases organic solvent tolerance and multiple antibiotic and heavy metal ion resistance in *Escherichia coli*. Appl Environ Microbiol 61: 2302-2307

4. Hayashi S, Aono R, Hanai T, Mori H, Kobayashi T, Honda H (2003) Analysis of organic solvent tolerance in *Escherichia* coli using gene expression profiles from DNA microarrays. J Biosc Bioeng 95: 379-383

5. Basak S (2013) Improving *E. coli* performance under stress by rewiring its global regulator Camp Receptor Protein (CRP). Dissertation. Nanyang Technological University, Singapore
