# Supplementary material for: Significantly improved solvent tolerance of Escherichia coli by global transcription machinery engineering
Source: Microb Cell Fact. 2015 Nov 5;14:175. doi: 10.1186/s12934-015-0368-4 (PMC4635540; doi:10.1186/s12934-015-0368-4)
Supplement: Supplementary file 6 — 10.1186/s12934-015-0368-4 Primers used in this study. [file 12934_2015_368_MOESM6_ESM.docx]

**Significantly improved solvent tolerance of *Escherichia coli* by global transcription machinery engineering**

**Additional files**

**Additional file 6**: Primers used in this study (Table S3).

**Table S3.** Primers used in this study

| Purpose | Primer | Sequence |
| --- | --- | --- |
| Amplification of *rpoD* | p-*rpoD*-F | 5'-AACCTAGGAGCTCTGATTTAACGGCTTAAGTGCCGAAGAGC-3' |
|  | p-*rpoD*-R | 5'-TGGAAGCTTTAACGCCTGATCCGGCCTACCGATTA-3' |
| Amplification of FRT-Kan-FRT | K-*yfgM*-F | 5'-GTAGCCGCGCATTTGCGCACGTTACTGGGTTAAGGAAGGAGAAGGACAGCGTGTAGGCTGGAGCTGCTTC-3' |
|  | K-*yfgM*-R | 5'-CGGAAAGCAGTCCTGGCAGCAGTAATTTACGCAATTGCATCGGGTCCCTCATTCCGGGGATCCGTCGACC-3' |
|  | K-*sdhB*-F | 5'-GCCCGGCATTCCCGCCGAAGATTCGTACTTACTAATGCGGAGACAGGAAAGTGTAGGCTGGAGCTGCTTC-3' |
|  | K-*sdhB*-R | 5'-CGGCACTGGTTGCCTGATGCGACGCTTGCGCGTCTTATCAGGCCTACGGTATTCCGGGGATCCGTCGACC-3' |
|  | K-*gapA*-F | 5'-GTAATTTTACAGGCAACCTTTTATTCACTAACAAATAGCTGGTGGAATATGTGTAGGCTGGAGCTGCTTC-3' |
|  | K-*gapA*-R | 5'-AAAAAAGAGCGACCGAAGTCGCTCTTTTTAGATCACAGTGTCATCTCAACATTCCGGGGATCCGTCGACC-3' |
|  | K-*pepB*-F | 5'-AATCCTGGGGCTGCCAACTGGCGGCCCTTTTACAAAGAAGGATAACTAAAGTGTAGGCTGGAGCTGCTTC-3' |
|  | K-*pepB*-R | 5'-CAGAATTTGTAGGCCGGATAAGGCGTTTACGCCGCATCCGGCATATTAGTATTCCGGGGATCCGTCGACC-3' |
|  | K-*bcp*-F | 5'-ATTAACGTCGTCAATTATTCCCAACATGATGAACAGGATGGAGTTAAGTA GTGTAGGCTGGAGCTGCTTC-3' |
|  | K-*bcp*-R | 5'-GTGAGGTATGCTGGCCGCAAGCGCAGCCAGCACGGAATGGAGCAAAGTAA ATTCCGGGGATCCGTCGACC-3' |
|  | K-*dppA*-F | 5'-TTTCCTTGAAAAAGTCAGGGATGCTGAAGCTTGGTCTCAGCCTGGTGGCTGTGTAGGCTGGAGCTGCTTC-3' |
|  | K-*dppA*-R | 5'-GAGCGCATCAGGCATTTTTGCCTTTGCCATCAGTCTTGTATGGCTTTTAAATTCCGGGGATCCGTCGACC-3' |
| Check for knockout | C-*yfgM*-F | 5'-AAACGGCAGTTGCGCAGGATAGC-3' |
|  | C-*yfgM*-R | 5'-ACAGCGAACAGCCGCTTAAAAGGGTAA-3' |
|  | C-*sdhB*-F | 5'-AAGCGTCAACATGGAACCGAAACTGC-3' |
|  | C-*sdhB*-R | 5'-CGGATAAGGCGTTCACGCCGCATC-3' |
|  | C-*gapA*-F | 5'- CCGCTTGACGCTGCGTAAGGTTTTT -3' |
|  | C-*gapA*-R | 5'-CGCAGTCCTTTAACTTCATTTTATCAGG-3' |
|  | C-*pepB*-F | 5'-TGCAACGCGAATCATTTAGCGGAAA-3' |
|  | C-*pepB*-R | 5'-GGGATTCGCAATTTGTTGAATTTG-3' |
|  | C-*bcp*-F | 5'- ACAGAACTCAATGCACAAGGCAGT -3' |
|  | C-*bcp*-R | 5'-CGAGAATGTGATCTTGATCACGAGAA-3' |
|  | C-*dppA*-F | 5'-TCACAATTGGAGCAGAATAATGCGTA-3' |
|  | C-*dppA*-R | 5'-GCAGAATTTTCGTAGGCCTGATAAGCG-3' |
|  | K_2_ | 5'-CGGTGCCCTGAATGAACTGC-3' |
|  | K_t_ | 5'-CGGCCACAGTCGATGAATCC-3' |
| Amplification of *yfgM*, *sdhB*, *gapA* and *pepB* for pQE-*yfgM*, pQE-sdhB, pQE-*gapA* and pQE-*pepB* constructions | E-*yfgM*-F | 5'-CGGGATCCATGGAAATTTACGAGAACGA-3' (*Bam*HI) |
|  | E-*yfgM*-R | 5'-CCCAAGCTTTCAGATGGACAAATTATTA-3' (*Hind*III) |
|  | E-*sdhB*-F | 5'-CGGGATCCATGAGACTCGAGTTTTCAATTT-3' (*Bam*HI) |
|  | E-*sdhB*-B | 5'-CCCAAGCTTTTACGCATTACGTTGCAACAA-3' (*Hind*III) |
|  | E-*gapA*-F | 5'-CGGGATCCATGACTATCAAAGTAGGTAT-3' (*Bam*HI) |
|  | E-*gapA*-R | 5'-CCCAAGCTTTTATTTGGAGATGTGAGCGA-3' (*Hind*III) |
|  | E-*pepB*-F | 5'-CGGGATCCATGACAGAAGCGATGAAGATTA-3' (*Bam*HI) |
|  | E-*pepB*-R | 5'-CCCAAGCTTTTACGCCGTTAACAGATTAGCTA-3' (*Hind*III) |
| use for RT-PCR | R-*yfgM*-F | 5'-GATTCTGCACGCTCCGCTT |
|  | R-*yfgM*-R | 5'-GTTCCAAAGAAGCCAGCGC |
|  | R-*sdhB*-F | 5'-AACGGCAAGAATGGTCTG |
|  | R-*sdhB*-R | 5'-GGTAAGGCTTAATTTTCTCA |
|  | R-*gapA*-F | 5'-TACATGGCATACATGCTGA |
|  | R-*gapA*-R | 5'-TTCGTCCCATTTCAGGTTAG |
|  | R-*pepB*-F | 5'-CATCAAGCATGTTCAGTTAA |
|  | R-*pepB*-R | 5'-GGTTATCCAGTTCCTGGC |
|  | R-*BCP*-F | 5'-ACAGCGTGTTCTGGTTTATT |
|  | R-*BCP*-R | 5'-TTATCGGTGCTGATACCCAG |
|  | R-*dppA*-F | 5'-TTTATTGCTCAGAAGGATCT |
|  | R-*dppA*-R | 5'-CCGATTTTAAATTCAACCAG |
|  | 16S rRNA-F | 5'-TTGCTCATTGACGTTACCCG |
|  | 16S rRNA-R | 5'-ACGCCCAGTAATTCCGATTA |

**References**

1. Ni Y, Song L (2013) Proteomic analysis of *Pseudomonas putida* reveals an organic solvent tolerance-related gene *mmsB*. PLoS ONE 8:e55858

2. Okochi M, Kurimoto M, Shimizu K, Honda H (2007) Increase of organic solvent tolerance by overexpression of *manXYZ* in *Escherichia coli*. Microbiol Biotechnol 73: 1394-1399

3. Nakajima H, Kobayashi K, Kobayashi M, Asako H, Aono R (1995) Overexpression of the *robA* gene increases organic solvent tolerance and multiple antibiotic and heavy metal ion resistance in *Escherichia coli*. Appl Environ Microbiol 61: 2302-2307

4. Hayashi S, Aono R, Hanai T, Mori H, Kobayashi T, Honda H (2003) Analysis of organic solvent tolerance in *Escherichia* coli using gene expression profiles from DNA microarrays. J Biosc Bioeng 95: 379-383

5. Basak S (2013) Improving *E. coli* performance under stress by rewiring its global regulator Camp Receptor Protein (CRP). Dissertation. Nanyang Technological University, Singapore
